# Do malpractice claim clinical case vignettes enhance diagnostic accuracy and acceptance in clinical reasoning education during GP training?

**DOI:** 10.1186/s12909-023-04448-1

**Published:** 2023-06-26

**Authors:** Charlotte van Sassen, Silvia Mamede, Michiel Bos, Walter van den Broek, Patrick Bindels, Laura Zwaan

**Affiliations:** 1 Department of General Practice, Erasmus Medical Center, Rotterdam, The Netherlands; 2 Institute of Medical Education Research Rotterdam (iMERR), Erasmus Medical Center, Rotterdam, The Netherlands; 3 Department of Psychology, Education and Child Studies, Erasmus School of Social and Behavioral Sciences, Rotterdam, The Netherlands

**Keywords:** Diagnostic errors, Medical education, Clinical reasoning education, Malpractice claim, Liability insurer, Clinical case vignette, General practice, Vocational training, Primary care, Education, Malpractice, Diagnostic Errors, Professional Misconduct, Missed Diagnosis, Medical Errors, Primary Care, General Practice, Family Practice

## Abstract

**Background:**

Using malpractice claims cases as vignettes is a promising approach for improving clinical reasoning education (CRE), as malpractice claims can provide a variety of content- and context-rich examples. However, the effect on learning of adding information about a malpractice claim, which may evoke a deeper emotional response, is not yet clear. This study examined whether knowing that a diagnostic error resulted in a malpractice claim affects diagnostic accuracy and self-reported confidence in the diagnosis of future cases. Moreover, suitability of using erroneous cases with and without a malpractice claim for CRE, as judged by participants, was evaluated.

**Methods:**

In the first session of this two-phased, within-subjects experiment, 81 first-year residents of general practice (GP) were exposed to both erroneous cases with (M) and erroneous cases without (NM) malpractice claim information, derived from a malpractice claims database. Participants rated suitability of the cases for CRE on a five-point Likert scale. In the second session, one week later, participants solved four different cases with the same diagnoses. Diagnostic accuracy was measured with three questions, scored on a 0–1 scale: (1) What is your next step? (2) What is your differential diagnosis? (3) What is your most probable diagnosis and what is your level of certainty on this? Both subjective suitability and diagnostic accuracy scores were compared between the versions (M and NM) using repeated measures ANOVA.

**Results:**

There were no differences in diagnostic accuracy parameters (M vs. NM next step: 0.79 vs. 0.77, *p* = 0.505; differential diagnosis 0.68 vs. 0.75, *p* = 0.072; most probable diagnosis 0.52 vs. 0.57, *p* = 0.216) and self-reported confidence (53.7% vs. 55.8% *p* = 0.390) of diagnoses previously seen with or without malpractice claim information. Subjective suitability- and complexity scores for the two versions were similar (suitability: 3.68 vs. 3.84, *p* = 0.568; complexity 3.71 vs. 3.88, *p* = 0.218) and significantly increased for higher education levels for both versions.

**Conclusion:**

The similar diagnostic accuracy rates between cases studied with or without malpractice claim information suggests both versions are equally effective for CRE in GP training. Residents judged both case versions to be similarly suitable for CRE; both were considered more suitable for advanced than for novice learners.

**Supplementary Information:**

The online version contains supplementary material available at 10.1186/s12909-023-04448-1.

## Background

Finding an explanation for patient’s health problems is one of the most important and complex tasks for general practitioners (GPs). GPs see a large volume of patients presenting with undifferentiated symptoms that could fit with common benign diseases as well as serious and uncommon diseases [1]. Moreover, GPs struggle with the balance between their fear of missing a serious disease on one hand, and the cost and scarcity of tests, that can even be harmful in case of over-testing, on the other hand. This, in combination with the fact that symptoms of a disease evolve over time, makes the diagnostic process complex and prone to error [1, 2]. It is estimated that diagnostic error, defined as a missed or delayed diagnosis [3], occurs in approximately 5% of all patients presenting in primary care [1, 3, 4], a figure amounting to a large number of patients that might be harmed each year. Improving clinical reasoning education (CRE) has been identified as a relevant way to reduce diagnostic error [5]. Currently in the GP vocational training, clinical reasoning skills are learned both in clinical practice and by practicing with examples, which are often fictitious, clinical case vignettes [6, 7]. Ideally, the content of case vignettes used in CRE reflects information that trainees need to know but have not yet mastered, for example because of insufficient exposure [8] or knowledge. This means that a wide variety of cases, reflecting the true complexity of clinical cases and contextual factors, often referred to as “situativity” [9, 10], is important in CRE [11]. In a previous study, we suggested using cases from a malpractice claim database to determine educational content, because these data reflect atypical presentations, contextual factors and knowledge gaps with a relevant impact on patients. The study showed that, in addition to more exposure to rare diseases, there is a need to include a greater diversity of common and uncommon diseases with atypical presentations, to expand illness scripts in physicians’ minds [12]. However, the way in which these malpractice claim cases should be presented in CRE is not yet clear. Specifically, it is not yet known whether adding information on an accepted malpractice claim to a clinical case facilitates or hinders learning.

Generally, elaborated case examples enjoy high acceptance among students [13, 14]. The integration of errors into them might make the case vignette more interesting, if perceived as a challenge [15]. In medical education, using erroneous examples is reported to improve the clinical skills [16, 17] and diagnostic knowledge [18–22] in students and residents. However, reading cases in which a medical error occurred, might also be perceived as a threat [18], especially in case the error resulted in a malpractice claim. This may trigger negative emotions [23–25] that can profoundly influence learning [26]. This has been studied extensively within educational and cognitive psychology [27–29], with inconsistent findings. Because cognitive resources are limited, emotional information evolutionarily takes precedence over neutral information, leading to deeper processing of emotional information [30, 31], which may hinder the processing of neutral information. Negative emotions are associated with systematic processing of information, leading to more detailed and item-specific processing than positive emotions, which are associated with heuristic processing of the information, leading to global and relational processing [32–34]. Moreover, negative affect reduces the ‘false memory effect’ [35] resulting in a better recall of negative items [23]. Negative information is therefore remembered more frequently, vividly and in more detail than neutral information [24–26, 30, 36–38]. Moreover, due to the ‘negativity bias’, people tend to pay more attention to and are more strongly influenced by the negative aspects of experiences [39, 40].

However, while some studies suggest that a (negative) emotional response facilitates memory and learning, others showed that an emotional response resulted in retaining less of the content. Emotions require working memory and create cognitive load, resulting in less working memory available for learning [41, 42]. This ‘cognitive load theory’ (CLT) has also been shown in medical education [43], where diagnostic performance of trainees in simulation training decreased as cognitive load increased [44–46]. Moreover, negative affect may lead to an over-dependency on familiar problem-solving strategies, which cannot be applied in every situation. In contrast, positive affect can increase cognitive flexibility, improving the transfer of clinical skills to new situations [36, 47].

Given these conflicting theories, we aimed to determine in this study whether knowing that a diagnostic error resulted in a malpractice claim, facilitated or hampered learning in future cases of the same disease in CRE. We determined whether there was a difference in diagnostic accuracy scores for diseases that participants were exposed to as an erroneous case either with or without stating that the case resulted in a malpractice claim. In addition, we compared the residents’ views on the suitability of the erroneous cases with or without a malpractice claim, which they diagnosed in the first phase of this study. While expert opinions on the position of CRE in the medical curriculum and CR curriculum design may be leading, learners’ opinions on the suitability of (the content of) case vignettes reflect motivation and should be taken into account, as motivational factors are relevant for successful learning. This allows us to make recommendations on the best way to use malpractice claim cases in case vignettes for CRE.

## Methods

All methods were carried out in accordance with relevant guidelines and regulations.

### Participants

Participants were first-year residents of the three-year GP vocational training at the department of General Practice at the Erasmus Medical Center in Rotterdam, The Netherlands.

### Setting

CRE during the post-graduate, three-year GP vocational training at Erasmus MC Rotterdam comprises eight themes, spread over the three years of training. Besides during daily clinical practice, supervised one-on-one by senior GPs, clinical reasoning is practiced during regular weekly one-day educational sessions, supervised by the department’s teaching staff. Each theme includes various fictive case vignettes on different diagnoses for trainees to solve.

### Study design

**Fig. 1:**
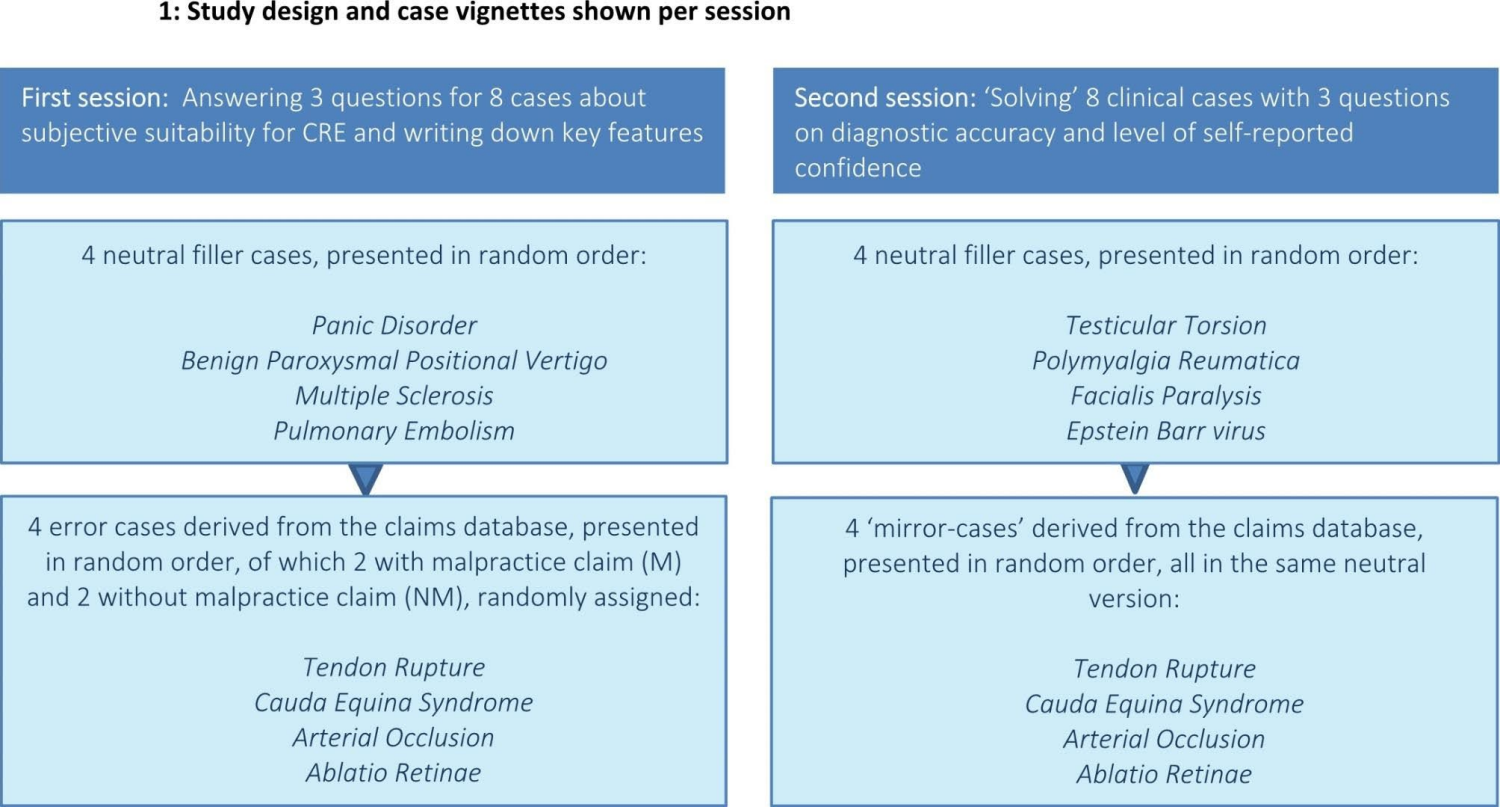
Study design and case vignettes shown per session

This study was a two-phased, within-subjects experiment. In the first session (learning phase), all participants were exposed to two erroneous cases with (M) and two erroneous cases without (NM) information on a malpractice claim, derived from a malpractice claims database (cases of interest), mixed with four neutral filler cases (all without malpractice claim). All participants were asked to answer questions about their opinion on suitability of the cases for CRE. In the second session (testing phase), one week later, all participants had to solve four different cases with the same diagnoses as the cases of interest (without malpractice claim information), again mixed with four neutral fillers. Participants were not aware of the aim of the research. Diagnostic accuracy for the cases of interest was scored with various parameters. Finally, a comparison was made for the various suitability- and diagnostic accuracy scores between the cases previously seen with or without a malpractice claim (see **Fig. **1).

### Materials and procedure

#### Claims database and case development

For this study, the liability insurance company covering 85% of the GP practices in the Netherlands made their anonymized claim database with cases filed between 2012 and 2017 available to us for educational and research purposes. The claim information was entered into a database and summarized by the insurance company, but the insurance company was not further involved in the analysis and interpretation of the study findings.

Eight different case files on four diagnoses were selected from the claims database by the principal investigators (two mirror cases of each diagnosis). Based on the complete malpractice claims files, clinical case vignettes were developed by two GPs (one study investigator and one independent lecturer from the department of general practice). For session one, two versions of the four cases were developed, namely a malpractice and non-malpractice version. Both versions of the cases were written in the same structure and with the same clinical and patient information, including the erroneous development during the diagnostic process. In all cases, the root of the error was a cognitive error, since these types of error are more relevant for the CR process than, for example, system-based errors. However, in the malpractice versions, the last sentence of the case stated that the error resulted in a malpractice claim and indemnity was paid, whereas the non-malpractice versions stated nothing concerning malpractice claim or indemnity paid. Besides the cases derived from the malpractice database, eight neutral fictive filler clinical case vignettes were developed for this study, which did not reflect any malpractice claims.

#### Procedure

This study consisted of two consecutive sessions, which took place one week apart in May-June 2020. Due to COVID-19 restrictions, both sessions took place online, embedded in the trainees’ regular educational program (temporarily online). For both sessions, the participants received a link to a Qualtrics questionnaire, which is a web-based survey tool. In order to prevent bias, during the sessions participants were not aware of the aim of the research, nor that the two sessions were linked.

#### First Session: learning and measuring subjective suitability for education

In the learning phase, participants were exposed to eight clinical vignettes. The first four vignettes, presented in random order, were fictive neutral filler cases to divert the attention away from erroneous cases and malpractice claims and to reduce the likelihood that participants realized that the two sessions were linked and some diagnoses of the two sessions were the same. The last four case vignettes were erroneous cases derived from the claims database (cases of interest), also presented in random order to prevent order effects. Each participant saw two erroneous cases with (M) and two erroneous cases without (NM) the malpractice claim ending. The participants were randomly assigned to one of the six variants of the questionnaire that were made to counterbalance the version of the case. Participants were asked to answer questions about their opinion on educational suitability for all eight cases with a Likert scale ranging from 1 to 5, where higher scores indicate a higher subjective suitability (see Table 1). To make sure the participants sufficiently examined the information on the clinical cases, they were also asked to note their key features of the eight cases directly after reading each full case. The correct diagnoses were given immediately for all cases.

**Table 1:** Questions for Subjective Suitability of Session 1. All answers on a Likert scale from 1 to 5 where 1 = not at all and 5 = very much

1. Do you think the case is overall appropriate for clinical reasoning education?
2. Is the complexity of the case overall high enough for clinical reasoning education? *Sufficient high complexity means that the disease can present in different ways or atypically and sufficient clinical reasoning is needed for diagnosing it. The disease should be well defined and it should not be a diagnosis per exclusionem.*
3. For which phase of medical education, you think the case is most appropriate? a. Bachelor b. Master c. GP vocational training

#### Second session: testing diagnostic accuracy and self-reported confidence

In the testing phase, the participants had to solve eight clinical cases, as a ‘regular’ clinical reasoning exercise. The first four case vignettes were new fictive neutral filler cases and the last four were the test cases, that is, mirror cases derived from the claim database to which the participants were exposed in the first session in either a malpractice version or a non-malpractice version. They were different cases with the same diagnoses as the first session. This time no diagnosis was given and all cases were presented neutrally, without error or malpractice claim. They started with four filler cases to prevent that the participants recognized the cases immediately from the first phase and in random order to prevent order effects. After reading the case, the participants were asked questions regarding their consideration of (differential) diagnoses and next steps, in order to be able to measure diagnostic accuracy (see Table 2). Answers could be further specified with free text or multiple choice (see **Appendix **1). In order to compare diagnostic accuracy between the cases previously seen ending with or without malpractice claim, the answers of the four mirror cases from the claims database (test cases) were subsequently scored and compared by the researchers.

**Table 2:** Questions for Diagnostic Accuracy of Session 2

1. What is your next step?
2a. What is your most probable diagnosis?
b. What is your level of certainty of your diagnosis?
3. What is your differential diagnosis?

### Analysis

Calculations were done using SPSS Statistics version 25 for Windows (IBM). Differences were considered significant at *p* < 0.05 level.

#### First Session: measuring subjective suitability for education

First, means for the answers of the subjective suitability-questions from Table 1 were computed per participant for the two cases of each version (M and NM). Subsequently, these means were compared using a repeated measures ANOVA. We took the variant of the questionnaire (1–6), that was used for counterbalancing the cases of interest, as a covariate.

Additionally, in order to assess whether subjective suitability differed significantly between educational levels, the subjective suitability scores were compared between the various educational levels for the two versions separately, using an ANOVA and a post hoc analysis with Bonferroni adjustment.

#### Second session: testing diagnostic accuracy and self-reported confidence

First, two senior GPs independently scored the accuracy of the answers to the questions of Table 2 on a three-point scale. Participants’ answers were scored as 1, 0.5 or 0 points, corresponding respectively to fully correct, partially correct, or incorrect answer. An interrater reliability analysis using the Kappa statistic was performed to determine consistency among raters. Disagreements among the evaluators were resolved by discussion.

Mean diagnostic accuracy scores for the two cases of each version (M and NM) were computed for all diagnostic accuracy parameters per participant. Subsequently, a repeated measures ANOVA was performed to compare the mean diagnostic accuracy scores between cases seen with and without a malpractice claim ending. Again, we took the variant of the questionnaire, that was used for counterbalancing the cases of interest, as a covariate. This was also done for self-reported confidence in most probable diagnosis. For the latter, a Pearson correlation coefficient for both versions was computed subsequently to assess the linear relationship between self-reported confidence and the accuracy score of ‘*most probable diagnosis’* to find out whether the participants’ diagnostic accuracy was calibrated with their self-reported confidence in their diagnosis. To assess the significance of the difference between the correlation coefficients of the malpractice versus the non-malpractice versions, a *z*-value was calculated with a Fisher *r*-to-*z* transformation. Linear regression was used to test whether the mean diagnostic accuracy score for *‘most probable diagnosis’* explained the mean confidence score.

## Results

### Participants

Of the total group of one hundred and fourteen residents, ninety-eight residents participated in this study, of which eighty-one participants (fifty-eight women (71,6%) and twenty-three men (28,4%), completed both questionnaires. Thirteen participants who attended the first session did not return to the second, three participants attended only the second session and four participants did not complete either questionnaire completely, therefore the final number of times in which each case has been seen in each version was not fully balanced (see Table 3 for randomization numbers).

**Table 3:** Number of Participants That Were Randomized over Versions of Cases in the First Session

Cases	n Malpractice	n Non-Malpractice
Tendon rupture	40	41
Cauda equina	41	40
Arterial occlusion	43	38
Ablatio retinae	38	43

### First session: measuring subjective suitability for education

Mean total duration of the first session was 38.7 min (SD 22.4 min). Assumptions of independence, normality and sphericity were met. The repeated measures ANOVA showed that there was no significant difference between malpractice and non-malpractice cases of the overall subjective suitability scores *F*(1.79) = 0.329 *p* = 0.568 and overall complexity scores *F*(1.79) = 1.545 *p* = 0.218. Regarding the questions of subjective suitability of the cases for the various levels of education, the repeated measures ANOVA showed no significant differences between malpractice and non-malpractice cases for all levels of education (Bachelor Level *F*(1.79) = 0.106 *p* = 0.745; Master Level *F*(1.79) = 0.486 *p* = 0.488; and GP Vocational Training Level *F*(1.79) = 0.015 *p* = 0.901) (see Table 4).

**Table 4:** Comparison of Various Mean Educational Suitability Scores Between Malpractice and Non-Malpractice Case Vignettes (Repeated Measures ANOVA) and Comparison of Suitability Scores Between Educational Levels for Both Case Versions (one-way ANOVA). Measured on a scale from 1 to 5, where a higher score indicates higher suitability

	Malpractice		Non-Malpractice		Repeated measures ANOVA
	Mean	SD	Mean	SD	*p*-value
**Overall Suitability**	3.68	0.93	3.85	0.88	0.568
**Overall Complexity**	3.71	0.90	3.88	0.94	0.218
**Suitability for Bachelor Level**	2.75	1.17	2.92	1.00	0.745
**Suitability for Master Level**	3.35	1.11	3.57	0.88	0.488
**Suitability for Vocational Training Level**	3.98	0.87	4.02	0.87	0.901
**One-way ANOVA ** ***p*** **-value**	< 0.001*a		< 0.001*b		

*significant

a. post-hoc Bonferroni: bachelor-master *p* = 0.001*; bachelor- GP vocational training *p* < 0.001*; GP vocational training-master *p* < 0.001*

b. post-hoc Bonferroni: bachelor-master *p* < 0.001*; bachelor- GP vocational training *p* < 0.001*; GP vocational training-master *p* = 0.007*

The scores of subjective suitability between the various educational levels were additionally compared with a one-way ANOVA for malpractice and non-malpractice cases separately, which showed a significant difference between subjective suitability scores of the Bachelor, Master and GP Vocational Training Levels for both case versions (malpractice *F*(2.240) = 27.689 *p* < 0.001; non-malpractice *F*(2.240) = 24.745 *p* < 0.001). For malpractice cases, Levenes test was significant, therefore a Welch test was performed as well, which showed the same results. Post hoc analysis with a Bonferroni adjustment revealed that for all cases suitability was significantly higher for higher education levels (see Table 4).

### Second session: testing diagnostic accuracy and self-reported confidence

Mean total duration of the second session was 47.1 min (SD 22.4 min). The interrater reliability was found to be moderate to substantial [48], Kappa = 0.636 (*p* < 0.001), 95% CI (0.558, 0.714) for most probable diagnosis; Kappa = 0.433 (*p* < 0.001), 95% CI (0.335, 0.531) for next step; and Kappa = 0.552 (*p* < 0.001), 95% CI (0.458, 0.646) for differential diagnosis. The cauda equina case was excluded from this calculation because after discussion between the reviewers, the diagnosis of herniated nuclei pulposi (HNP) should also be considered a correct answer, which was not taken into account in the independent scoring and which comprised the majority of answers.

In Table 5, overall mean diagnostic accuracy scores as well as mean scores per case are presented for both malpractice and non-malpractice versions. Assumptions of independence, normality and sphericity were met. A repeated measures ANOVA showed that there was no significant difference in the various mean diagnostic accuracy scores between malpractice – and non-malpractice cases: ‘*What is your next step*’ *F*(1.79) = 0.448 *p* = 0.505, ‘*What is your differential diagnosis’ F*(1.79) = 3.318 *p* = 0.072 *and ‘What is your most probable diagnosis’ F*(1.79) = 1.555 *p* = 0.216. Furthermore, there was no significant difference in the self-reported confidence scores (0-100%) for the answer for *‘What is your most probable diagnosis’* between the two versions of cases *F*(1.79) = 0.747 *p* = 0.390 (see Table 6).

**Table 5:** Comparison of Mean Scores on Various Diagnostic Accuracy Parameters between Malpractice and Non-Malpractice Cases and Mean Diagnostic Accuracy Scores Per Case. Mean scores range from 0–1 points

		Score Malpractice			Score Non-Malpractice			Repeated measures ANOVA
		N	Mean	SD	N	Mean	SD	*p*-value
**Next step**	*Overall*	*81*	*0.79*	*0.26*	*81*	*0.77*	*0.26*	*0.505*
	Tendon rupture	40	0.66	0.46	41	0.59	0.47	
	Cauda equina/HNP*	41	1.00	0.00	40	0.98	0.16	
	Arterial occlusion	43	0.81	0.39	38	0.79	0.41	
	Ablatio retinae	38	0.68	0.34	43	0.74	0.30	
**Differential diagnosis**	*Overall*	*81*	*0.68*	*0.29*	*81*	*0.75*	*0.27*	*0.072*
	Tendon rupture	40	0.66	0.47	41	0.65	0.48	
	Cauda equina/HNP*	41	0.95	0.22	40	0.93	0.27	
	Arterial occlusion	43	0.50	0.45	38	0.63	0.43	
	Ablatio retinae	38	0.59	0.49	43	0.80	0.40	
**Most probable diagnosis**	*Overall*	*81*	*0.52*	*0.33*	*81*	*0.57*	*0.31*	*0.216*
	Tendon rupture	40	0.51	0.50	41	0.52	0.50	
	Cauda equina/HNP*	41	0.83	0.38	40	0.89	0.31	
	Arterial occlusion	43	0.40	0.44	38	0.42	0.44	
	Ablatio retinae	38	0.34	0.47	43	0.44	0.49	

*The high score for cauda equina can be explained by the fact that based on the case description, the diagnosis herniated nuclei pulposi (HNP) also had to be considered a correct answer. As a result, many answers were scored correct. Excluding this case did not significantly alter the results of the statistical tests

**Table 6:** Comparison of Mean Self-Reported Confidence for ‘Most Probable Diagnosis’ between Malpractice and Non-Malpractice Cases. Mean self-reported confidence scores range from 0-100%

	Malpractice		Non-Malpractice		Repeated measures ANOVA
	**Mean**	**SD**	**Mean**	**SD**	***p*** **-value**
**Mean self-reported confidence score in ‘Most probable diagnosis’**	53.69	16.65	55.81	16.15	0.390

There was a significant and positive Pearson correlation between the accuracy score of *‘What is your most probable diagnosis’* and the self-reported confidence in this answer for both malpractice *r*(79) = 0.363 *p* < 0.001 and non-malpractice cases *r*(79) = 0.261 *p* = 0.019. There was no significant difference in the correlation coefficients between malpractice and non-malpractice cases, calculated by the Fisher *r*-to-*z* transformation, *z* = 0.71 *p* = 0.477 (two-tailed) (see Table 7).

**Table 7:** Correlation Between Mean Self-Reported Confidence and Mean Diagnostic Accuracy Score for ‘Most Probable Diagnosis’ for Malpractice and Non-Malpractice Cases

	Malpractice	Non-Malpractice	Difference *r*_malpractice_-*r*_non−malpractice_ Fisher *r*-to-*z*
**Pearson Correlation (** ***r*** **)**	0.363	0.261	*z* = 0.71
***p*** **-value**	< 0.001*	0.019*	0.477

*significant

**Fig. 2:**
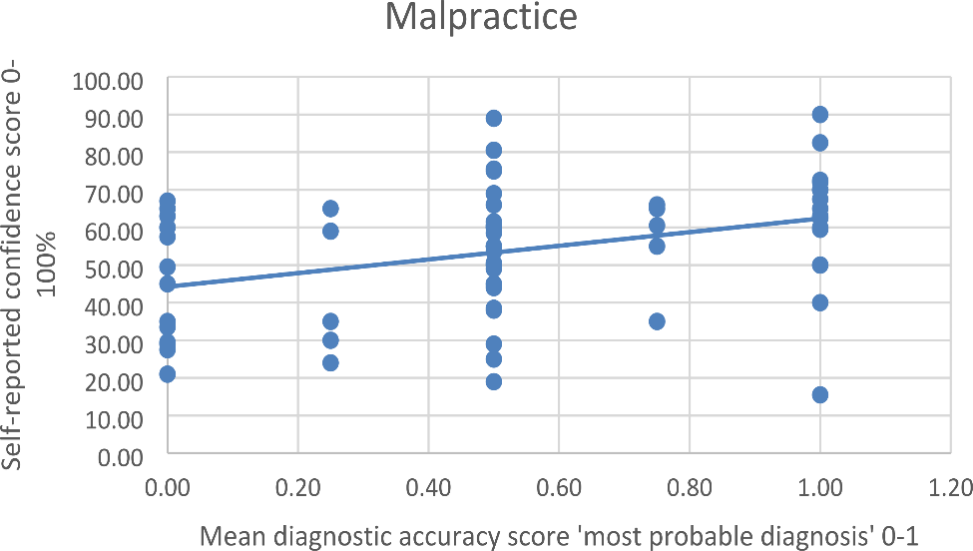
Linear Regression of Mean Diagnostic Accuracy Score ‘Most Probable Diagnosis’ and Self-Reported Confidence of Malpractice Cases

**Fig. 3:**
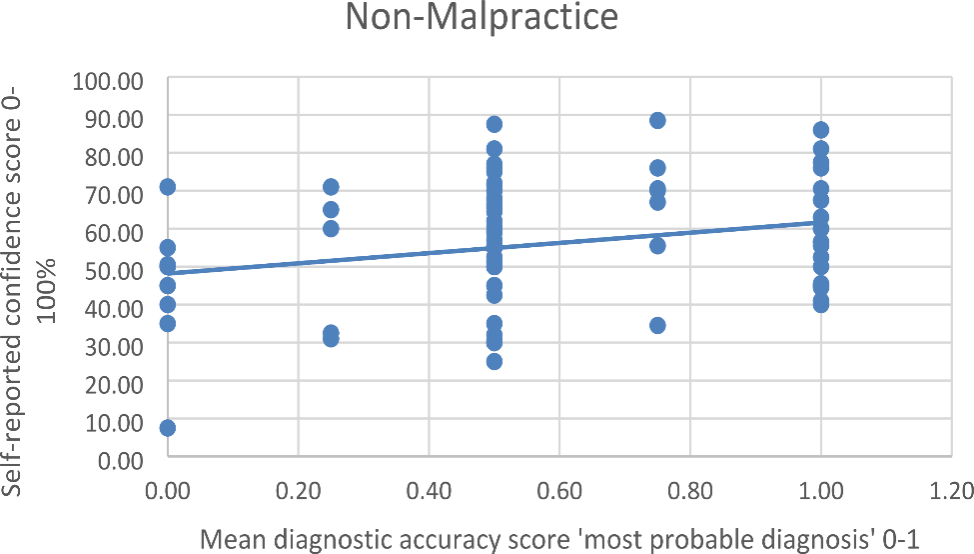
Linear Regression of Mean Diagnostic Accuracy Score ‘Most Probable Diagnosis’ and Self-Reported Confidence of Non-Malpractice Cases

The results of the simple linear regression indicated that the mean diagnostic accuracy score of ‘*What is your most probable diagnosis’* significantly explained 13.19% of the variation in the confidence score for malpractice cases *F*(1.79) = 12.008 *p* < 0.001. The regression coefficient indicated that an increase of 0.10 points in the diagnostic accuracy score corresponded to an average increase in confidence of *B* = 18.137% for malpractice cases (see Fig. 2). For non-malpractice cases, 6.82% of the variance in the confidence score could be explained by the diagnostic accuracy score *F*(1.79) = 5.782 *p* = 0.019 with a regression coefficient of *B* = 13.444% (see Fig. 3), which was also statistically significant.

## Discussion

As previously suggested in the literature, malpractice claim databases may provide an unique opportunity for deriving educational benefit from the mistakes from others [49, 50]. Malpractice claims could add situativity to CRE by enriching and supplementing the CR curriculum for advanced learners with a variety of clinical case examples with atypical disease presentations and complex contextual factors [12], thereby expanding illness scripts in physicians’ minds and improving diagnostic performance [51–54]. This study examined whether knowing that a medical error resulted in a malpractice claim affects diagnostic accuracy after one week on future cases of the same disease in CRE in first-year GP residents. The results showed no significant differences in the scores for the various parameters of diagnostic accuracy and self-reported confidence between cases previously seen with malpractice claim information versus cases without malpractice claim information. Participants’ reported subjective suitability scores of using erroneous cases for CRE were also similar for malpractice- and non-malpractice versions, and both case versions were considered more suitable for advanced learners than for novice learners.

Based on these findings, it seems that knowing whether an erroneous case resulted in a malpractice claim neither facilitates nor hampers learning and performance, compared to erroneous cases without malpractice claim information. Negative emotions triggered by the information of malpractice claim could have enhanced retention in memory of the case examples seen in the first session [23–26, 30, 32–40]. On the other hand, if the malpractice claim information would increase cognitive load, adverse effects on retention could be expected [41–47]. However, our findings do not support any of these possibilities.

Therefore, erroneous cases with and without malpractice claim information seem equally effective for CRE. A caution is that the use of malpractice claims in CRE would possibly be better balanced with the use of erroneous cases without malpractice claims and neutral cases to avoid possible negative side effects such as overdiagnosis and overtesting.

Because the emotional impact of reading the cases in this study was not measured, we could not quantify the intensity of the emotional response, if any, and compare cases with and without malpractice claims. However, since students show an intense emotional response to the idea of making errors in patient care and report that medical errors that cause serious harm to the patient in particular impress them [50], it is likely that adding an error or malpractice claim to a case vignette and reading contextual information and the impact of the error on the patient (situativity) can create emotional valence in a case vignette.

The absence of a difference in diagnostic accuracy scores between the malpractice and non-malpractice versions, could have been caused by the fact that due to the within-subjects design of the study, all participants were exposed to *erroneous* cases, which in itself may have induced an emotional response. Furthermore, to prevent information bias between the non-malpractice and malpractice case vignettes, the malpractice case vignettes were stripped of personal context of the impact of the claim on the patient. As a result, participants might have had only a minor emotional response on reading solely the ‘stripped’ statements on the malpractice claims, further decreasing the difference in emotional response between the case versions. This limited response was possibly compounded by the fact that the participants in this study were first-year GPs in training and still under the strict responsibility of their daily supervisor. Since vocational training lasts a total of three years, it is possible that the residents did not yet feel the “urgency” of their own responsibility for error.

Besides these arguments for a weak emotional response explaining to the lack of differences in the results, a cognitive factor may have contributed as well. Since the participants did not have to solve the case in the learning phase but answer subjective suitability questions only, this might have led to shallow cognitive processing of the diagnosis.

Another possible explanation for the lack of difference could be that, although processing the emotional claim information might have come (partly) at the expense of clinical information, as found in previous studies [55–57], it did not affect diagnostic accuracy scores in our participants, because advanced learners such as our participants might be able to handle more cognitive load since they have already mastered the basics. A similar theory is confirmed by the studies of Große and Renkl [58, 59], which show that especially learners with solid knowledge profit from incorrect examples. In addition, several studies show that enrichment of case vignettes with atypical disease presentations, rare conditions or complex contexts is especially beneficial for advanced students [42, 60–62]. In line with these findings, it might be beneficial for advanced learners to add impressive emotional information such as malpractice claims to a case vignette to add an extra dimension to the reasoning process and thus intensify it, as extra training. However, more research is needed to quantify the effects of adding malpractice claims statements to case vignettes on the learners’ emotion and type of information remembered from claim case vignettes, besides the current study on diagnostic accuracy. In addition, further research needs to determine what level of learner expertise is most appropriate to achieve equivalent or even positive effects on learning of adding claim information, compared to neutral cases.

The equal scores in self-reported confidence between the two case types suggests that reading about a malpractice claim did not affect self-reported confidence, for example by evoking negative emotions such as fear, anxiety or uncertainty that could undermine self-confidence and hinder learning. The small but positive correlation between the most probable diagnosis and self-reported confidence for both case versions indicates that self-reported confidence was poorly calibrated with correct diagnoses. This finding is consistent with the literature on miscalibration and overconfidence [63–67].

Residents’ evaluation of overall subjective suitability- and complexity-levels for using erroneous malpractice and non-malpractice cases for clinical reasoning education showed that malpractice erroneous cases were considered equally suitable as non-malpractice erroneous cases for clinical reasoning education. These results are in contrast with the unofficial reports of our GP residents, who indicate that they find malpractice cases more interesting and claim to remember them better than regular cases. Subjective suitability scores for both malpractice and non-malpractice cases increased significantly with increased level of learners. These subjective evaluations support the theory that clinical reasoning in undergraduate medicine should be introduced with low-complex case vignettes with typical presentations for the development of basic medical knowledge. For more advanced students it should be promoted with more complex, rare or atypical case presentations, as reflected in erroneous cases that have inherent clinical and contextual complexity, and maybe even with impressive emotional information such as malpractice claims, that require a deeper level of reasoning and understanding and more cognitive flexibility [42, 60–62].

### Limitations

Besides the earlier mentioned arguments possibly explaining the absence of a difference in diagnostic accuracy scores between the non-malpractice and malpractice cases, this study had several methodological limitations.

First, our Kappa statistic for scoring diagnostic accuracy was lower than found in previous studies [45, 55, 68, 69]. This is probably because, unlike previous studies, we did not train the evaluators beforehand. Our two GP evaluators first independently scored all responses with only a general introduction, followed by a consensus discussion. Although this reduced the Kappa value, it encouraged discussion of the correctness of the answers and contributed to careful scoring of diagnostic accuracy. We measured diagnostic accuracy not only by the accuracy of the final diagnosis, but also by the ‘next step’ and ‘differential diagnosis’ parameters to account for the process of arriving at the correct diagnosis [70].

Second, the within-subjects design may have induced a carry-over effect of the malpractice cases on the non-malpractice cases. A between-group analysis with an added neutral version would be appropriate not only to overcome this problem, but also the problem of the carry-over effect created by exposing all participants to erroneous cases, which may have induced emotional response in itself.

Furthermore, we conducted our study only among first-year general practitioners in training, meaning that our results may be not applicable to undergraduate students, graduate students or more experienced residents or physicians. Further research is needed to understand whether diagnostic accuracy scores for malpractice and non-malpractice claim cases depend on differences in prior clinical experience among residents and whether they are generalizable among residents of other specialties. Moreover, our study was conducted at a single academic university in the Netherlands. In our institute, a significant amount of time is spent teaching clinical reasoning and our students are therefore relatively well trained in clinical reasoning. This might limit generalizability to sites with less focus on developing clinical reasoning abilities. In addition, cultural differences in dealing with diagnostic errors and claims and the associated emotions were not considered in this study. It is therefore recommended that this study be expanded to other parts of the world. Finally, thirteen residents participated in session 1 but not in session 2 of the study, and four residents did not complete the questionnaire for one of the sessions. Because of ethics committee requirements and informed consent guidelines for participants in scientific research, participants had the right to drop out without giving reasons, therefore we had no insight into the reasons for these dropouts.

## Conclusion and recommendations

This study shows that knowing that a diagnostic error resulted in a malpractice claim has no impact on diagnostic accuracy of future clinical cases of the same diagnosis or self-reported confidence in first-year GP residents. Residents’ opinions on suitability of both malpractice- and non-malpractice versions of erroneous cases for CRE were also similar and increased for both versions with higher levels of education. This indicates that both types of erroneous cases were considered more appropriate for advanced than for novice learners. Based on these findings, it seems that a description of the malpractice claim itself could be added to a clinical case vignette for GP residents. To make more specific recommendations on the best way to present malpractice claim cases for improving clinical reasoning education, more detailed research is needed. We recommend further research with a between-groups analysis with an added neutral version not only on diagnostic accuracy, but also on the quantification of the emotional response triggered by malpractice cases compared to solely erroneous- and neutral cases. In addition, the differences in how information is processed and what kind of information is remembered from different types of cases should be further quantified. This could be done for different levels of learners and in different parts of the world.

## Electronic supplementary material

Below is the link to the electronic supplementary material.

Supplementary Material 1

## Data Availability

The datasets used and/or analysed during the current study are available from the corresponding author on reasonable request.

## List of abbreviations

CRE: Clinical Reasoning Education
GP: General practitioner
M: Malpractice
NM: Non-Malpractice
