# Supplementary material for: Do malpractice claim clinical case vignettes enhance diagnostic accuracy and acceptance in clinical reasoning education during GP training?
Source: BMC Med Educ. 2023 Jun 26;23:474. doi: 10.1186/s12909-023-04448-1 (PMC10294315; doi:10.1186/s12909-023-04448-1)
Supplement: Supplementary file 1 — Supplementary Material 1 [file 12909_2023_4448_MOESM1_ESM.docx]

**Appendix 1**

**Questionnaire of the second session**

1. *What is your next step?*
   1. Advice and wait
   2. Additional diagnostic testing – laboratory (blood, urine, microbiology), radiology (x-rays, ultrasound, MRI, CT) etc
      1. Please specify the diagnostic tests you would advice *free text*
   3. Referral – specialist care, paramedic care, etc
      1. Please specify to whom you would refer *free text*
         1. In what period?
            1. Directly
            2. Urgent within 1-2 days
            3. Within 2 weeks
            4. Whenever possible
            5. Other, namely *free text*
   4. Treatment
      1. Please specify which treatment you would advice *free text*
   5. Other, namely *free text*
2. *What is your most probable diagnosis?* *free text*
   1. *What is your level of certainty on your most probable diagnosis?* Scale 0-100%
3. *What are your differential diagnoses?* *free text*
